# Sexually-transmitted hepatitis C virus reinfections among people living with HIV in Taiwan: the emerging role of genotype 6

**DOI:** 10.1080/22221751.2022.2065933

**Published:** 2022-04-12

**Authors:** Guan-Jhou Chen, Hsin-Yun Sun, Sui-Yuan Chang, Li-Hsin Su, Yi-Ting Chen, Szu-Min Hsieh, Wan-Da Liu, Wang-Huei Sheng, Yu-Shan Huang, Kuan-Yin Lin, Yi-Ching Su, Wen-Chun Liu, Chien-Ching Hung

**Affiliations:** a Department of Internal Medicine, National Taiwan University Hospital and National Taiwan University College of Medicine, Taipei, Taiwan; b Min-Sheng General Hospital, Taoyuan, Taiwan; c Department of Clinical Laboratory Sciences and Medical Biotechnology, National Taiwan University College of Medicine, Taipei, Taiwan; d Department of Laboratory Medicine, National Taiwan University Hospital and National Taiwan University College of Medicine, Taipei, Taiwan; e Department of Tropical Medicine and Parasitology, National Taiwan University College of Medicine, Taipei, Taiwan; f Department of Medical Research, China Medical University Hospital, Taichung, Taiwan; g China Medical University, Taichung, Taiwan

**Keywords:** Men who have sex with men, sexually transmitted disease, condomless receptive anal intercourse, genotype switch, phylogenetic analysis

## Abstract

Hepatitis C virus (HCV) reinfections after successful treatment with direct-acting antivirals (DAAs) pose a significant challenge to HCV elimination, especially among high-risk people living with HIV (PLWH). In this study, PLWH who had achieved HCV viral clearance with DAAs were included between January 2018 and June 2021. PLWH having acquired HCV infections after 2017 were classified as “recent-infection group,” and those before 2017 as “remote-infection group,” and the incidences of HCV reinfection were compared between two groups. Clinical and behavioural characteristics were evaluated to identify associated factors with HCV reinfection. A total of 284 PLWH were included: 179 in the recent-infection group and 105 in the remote-infection group. After a median follow-up of 2.32 years (interquartile range [IQR], 0.13–3.94), the overall incidence of HCV reinfection was 5.8 per 100 person-years of follow-up (PYFU). The incidence in the recent-infection group was significantly higher than that in the remote-infection group (9.8 vs. 0.4 per 100 PYFU, *p* < 0.001). The leading HCV genotypes before DAA treatment were genotypes 2 (31.0%), 1b (26.8%), and 6 (21.8%); however, genotype 6 (58.8%) became predominant upon reinfection. Younger age (adjusted odds ratio [aOR] per 1-year increase, 0.95; 95% CI, 0.90–0.99), condomless receptive anal sex (aOR, 14.5; 95% CI, 2.37–88.8), rimming (aOR, 3.87; 95% CI, 1.14–13.1), and recent syphilis (aOR, 2.73; 95% CI, 1.26–5.91) were linked to HCV reinfections. In conclusion, PLWH acquiring HCV after 2017 had a significantly higher risk for sexually-transmitted HCV reinfections. The predominance of HCV genotype 6 reinfections suggests possible on-going clustered HCV infections among at-risk PLWH.

## Introduction

In recent years, the development of direct-acting antivirals (DAAs) has revolutionized the treatment of acute and chronic hepatitis C. DAAs are much more tolerable and effective than interferon-based therapy. Studies have demonstrated that DAAs achieve a very high rate of sustained virologic response (SVR), which is subsequently associated with reduced all-cause mortality, liver-related complications and hepatocellular carcinoma [1–3]. Moreover, the widespread use of DAAs could also reduce circulating hepatitis C virus (HCV) in the community and prevent incident HCV infections [4]. In the United Kingdom, a study in 2020 showed that HCV incidence had reduced by 68% since the epidemiological peak in 2015 [5]. Similarly, acute HCV infection among HIV-positive men who have sex with men (MSM) was reduced by 51% with unlimited access to DAAs in the country [6]. With these clinical evidence, the World Health Organization (WHO) is now promoting HCV testing and DAA treatment, to reduce new cases of viral hepatitis by 90% and hepatitis-related deaths by 65% by 2030.

Despite the effectiveness of DAAs, the control and elimination of HCV are still challenging. In 2015, WHO estimated that 71 million people were suffering from chronic hepatitis C globally, with 1.7 million incident cases that year [7]. The global estimates in 2021 showed only a slightly reduced case number of chronic hepatitis C to 58 million and that of new infections to 1.5 million [8]. In resource-limited settings, access to testing and DAAs remains the most significant obstacle to HCV elimination [9]. On the other hand, in countries with wider access to HCV testing and DAAs, investigators have observed high rates of HCV reinfection after successful clearance of HCV among high-risk populations [10–16].

For people living with HIV (PLWH), many epidemiological studies have demonstrated high rates of HCV coinfection due to the shared risk factors, such as sharing needles, syringes and diluent or unprotected sexual contacts [10]. In clinical studies focusing on HIV-positive MSM in different countries, the annual rates of HCV reinfection after attaining SVR ranged from 1.9% to 21.8% [11–16]. Major clinical guidelines now recommend periodic testing for HCV RNA to monitor potential HCV reinfections among high-risk populations [17,18]. Therefore, understanding the epidemiology of HCV reinfection among these high-risk populations should be considered an integral part of the programme of HCV elimination to achieve the WHO 2030 targets.

In this study, we aimed to estimate the rate of HCV reinfection in the era of improved access to DAAs, identify factors associated with HCV reinfection, and describe the genetic linkage between these cases of HCV reinfection.

## Materials and methods

### Study setting and design

In Taiwan, the National Health Insurance (NHI) started to reimburse DAAs for patients with chronic HCV in 2017. A previous study revealed that the annual rate of HCV reinfection among PLWH who attained SVR by either DAAs or interferon-based regimens was 8.2 per 100 person-years of follow-up (PYFU) (95% confidence interval [CI], 5.2–13.1) [19]. In 2019, to achieve the WHO 2030 targets, the Taiwanese NHI broadened the coverage of DAAs to include all patients with HCV viremia, regardless of their chronicity. The second course of DAA treatment can be reimbursed for those with HCV relapses or reinfections following the first course of DAA treatment in early 2021 [20].

This was a single-centre, retrospective cohort study conducted at the National Taiwan University Hospital (NTUH). PLWH with confirmed HCV viremia were included if they had completed DAA therapy and the end-of-treatment (EOT) assessment between 1st January 2018 and 30th June 2021. In this study, successful treatment with DAA was defined as those who had achieved SVR 12 weeks after EOT; or those who had undetectable HCV RNA at EOT but had a rebound of HCV viremia with genotype switch before attaining SVR assessment. PLWHs who did not meet these two definitions were considered to have DAA treatment failure and were excluded from our study. Those who were lost to follow-up after the completion of DAA were also excluded.

In this study, we hypothesized that PLWH who had recently acquired HCV were more likely to continue their high-risk behaviours after DAA treatment. Therefore, the included PLWH were classified as “recent-infection group” and “remote-infection group” according to their timing of HCV infection. The recent-infection group comprised those who had seroconversion of anti-HCV antibodies after 2017 when NHI in Taiwan started to reimburse DAAs; or those who had new episodes of HCV viremia after 2017. On the other hand, those who had HCV viremia before 2017 and those who had indeterminate chronicity of HCV viremia were classified as the remote-infection group. Participants in both groups were followed from the EOT of DAA treatment until their first confirmed HCV viremia, death, loss to follow-up, or the end of the observation period (defined as 31st December 2021), whichever occurred first.

Medical records of all included PLWH were reviewed retrospectively to collect information on demographic and clinical characteristics. According to the national HIV treatment guidelines in Taiwan, PLWHs were followed at the hospital every 3 months, and laboratory tests were performed every 3–6 months, including plasma HIV RNA load (PVL), CD4 cell count, renal function, liver function, lipid profile and serum glucose level. The Research Ethics Committee of NTUH approved the study design (registration numbers: 201605103RINC and 201605128RINC) and waived the need for informed consent for the retrospective collection of clinical information.

### Laboratory investigations of HCV infection during follow-up

To detect HCV reinfection, PLWH underwent testing for plasma HCV RNA periodically after completing their DAA treatment. During the observation period between June 2019 and December 2021, pooled plasma HCV RNA testing was performed every 3 months to detect HCV viremia [21]. In short, plasma samples from 20 individuals were pooled together for HCV RNA testing, and subsequent testing of mini-pools consisting of 5 samples would be performed if the initial testing was positive; and testing of individual samples would be performed for the mini-pooled samples tested positive for HCV RNA. Those who were not included in the pooled plasma HCV RNA testing also tested for HCV RNA every 3–6 months after completing DAA treatments according to the Taiwanese HIV treatment guidelines. The plasma HCV RNA and genotyping were performed using the Roche Cobas 6800 system (AmpliPrep HCV Test, v2.0, Roche, USA), with a detection limit of 15 IU per milliliter.

For PLWH who participated in the pooled-plasma HCV RNA testing study and had their plasma samples collected during the observation period, phylogenetic analysis was performed to determine the genetic relatedness with HCV *NS5B* gene sequences. DNA sequencing of a 366-base fragment covering partial *NS5B* gene (nucleotides 8294–8629 of HCV reference strain H77) was amplified by the polymerase-chain reaction. Sequences obtained were then aligned using the MUSCLE algorithm [22]. The tree was then constructed by the neighbour-joining method with the Tajima-Nei pairwise distances in the MEGAX software (version 10.1.8). The time scale of phylogenetic tree was constructed using the best-fitting root using the heuristic residual mean squared function of TempEst software (version 1.5.3).

### Questionnaire interviews for behavioural characteristics

PLWHs who participated in the pooled-plasma HCV RNA testing study were also invited to participate in a face-to-face questionnaire interview to inquire into the behaviours that might increase the risk of acquiring HCV, including the use of illicit or injecting drugs, unsafe sexual practices, number of sexual partners, and the frequency of condom use in sexual contacts. The study of pooled plasma HCV RNA testing and behavioural questionnaire interviews were approved by the Research Ethics Committee of NTUH (registration number: 201904086RIPB), and each participant in this substudy provided written informed consent.

### Statistical analysis

Comparisons of demographic and clinical characteristics were made between the recent- and remote-infection groups. Non-categorical variables were compared using Student’s *t*-test or Mann–Whitney *U-*test, and categorical variables were compared using the chi-square test or Fisher’s exact test. The incidences of HCV reinfection in the recent- and remote-infection groups were calculated separately and compared.

To identify potential factors associated with HCV reinfections, we constructed two multivariate logistic regression models with different demographic, clinical and behavioural factors. The first multivariate model included all PLWH and assessed the association between different demographic or clinical factors and the subsequent occurrences of HCV reinfection. A backward elimination process was used during the multivariate analyses, in which all possible demographic and clinical factors were included in the model initially, and factors were removed from the model, starting with factors with the largest *p*-value. The process was repeated until all factors in the model had a *p*-value of <0.2. In the second model, only PLWH who participated in questionnaire interviews were included, and we started with the behavioural risk factors and the relevant clinical factors in the previous model. A similar backward elimination process was performed until all factors in the model had a *p*-value of <0.2. All statistical analyses were performed using STATA software v.14.0 S/E (StataCorp LP, College Station, TX). All *p*-values were two-sided.

## Results

**Figure 1.**
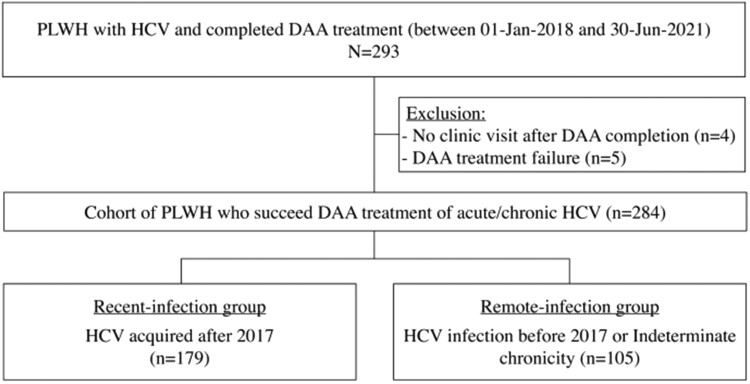
Study flow. Abbreviations: PLWH, people living with HIV; HCV, hepatitis C; DAA, direct-acting antivirals.

During the 4-year study period, 293 PLWH received DAAs for HCV infection at NUTH. A total of 284 PLWH who had successfully treated with DAAs were included in our study, with 179 being infected with HCV after 2017 (the recent-infection group) and 105 being otherwise classified in the remote-infection group (Figure 1). The recent-infection group had contributed 335.87 PYFU with a median follow-up of 1.7 years (interquartile range [IQR], 1.2–2.6), while the remote-infection group contributed 250.32 PYFU (median, 2.5 years; IQR, 2.2–2.7).

**Figure 2.**
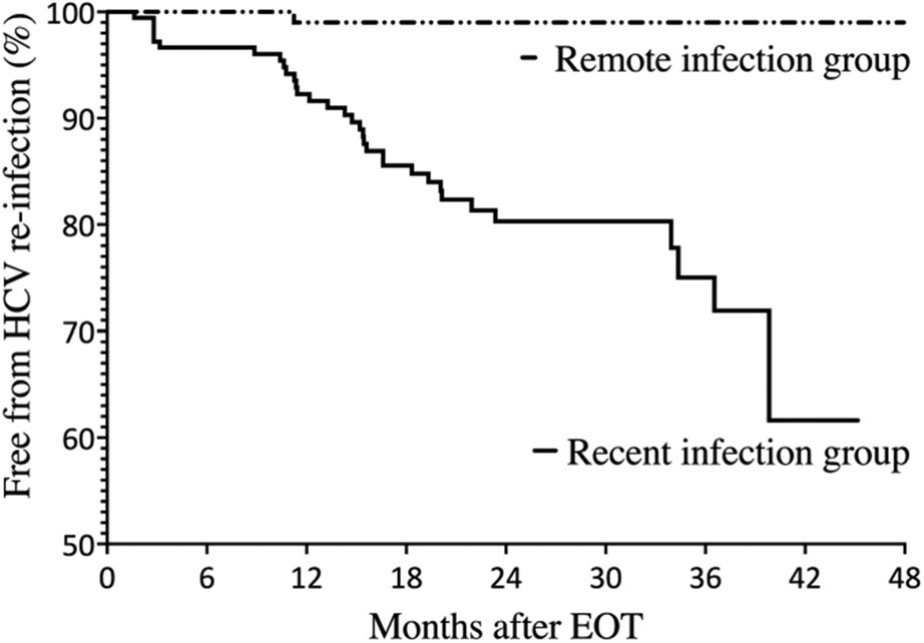
Kaplan-Meier estimates showing the cumulative rates of hepatitis C reinfection after completing DAA treatments for the recent-infection group and remote-infection group. Abbreviations: HCV, hepatitis C virus; EOT, end-of-treatment.

Baseline characteristics of the two groups are shown in Table 1. Compared to those in the remote-infection group, PLWH in the recent-infection group were significantly younger (mean age, 36.6 vs. 42.2 years); more likely to have recent syphilis within 3 months (45.3% vs. 18.1%), to be MSM (89.9% vs. 72.4%) and to have received multiple courses of HCV treatments (12.8% vs. 2.9%); and had shorter follow-up duration (median, 1.7 vs. 2.5 years). For those who participated in the behavioural questionnaire interviews, there was no statistically significant difference in terms of risky sexual practices between these two groups (Table 1). During the observation period, 34 episodes of HCV reinfection were documented in our cohort, with 33 cases in the recent-infection group and 1 case in the remote-infection group. The overall incidence rate of HCV reinfection for the entire cohort was 5.8 per 100 PYFU (95% CI, 4.1–8.1). The incidence was significantly higher in the recent-infection group (9.8 per 100 PYFU; 95% CI, 7.0–13.8) than in the remote-infection group (0.4 per 100 PYFU; 95% CI, 0.1–2.83) (*p *< 0.001). In the Kaplan-Meier survival analysis, there was also a significant difference between the two groups (Figure 2).

**Table 1.** Baseline Characteristics of the included people living with HIV who had HCV coinfection.

Baseline Characteristics	Recent-infection group (*n* = 179)	Remote-infection group (*n* = 105)	*p*-value
Age, mean (SD), years	36.6 (7.8)	42.2 (10.5)	<0.001
Male sex, *n* (%)	179 (100)	102 (97.1)	0.05
Risk group for HIV infection			<0.001
Men who have sex with men, *n* (%)	161 (89.9)	76 (72.4)	
Injecting drug users, *n* (%)	11 (6.1)	19 (18.1)	
Having received multiple (≥2) courses of HCV treatments prior to this study, *n* (%)	23 (12.8)	3 (2.9)	<0.001
Duration of follow-up, median (IQR), years,	1.7 (1.2–2.6)	2.5 (2.2–2.7)	<0.001
Syphilis within 6 months, *n* (%)	81 (45.3)	19 (18.1)	<0.001
HBsAg positivity, *n* (%)	16 (8.9)	8 (7.6)	0.44
Baseline plasma HIV RNA load, median (IQR), log_10_ copies/ml	1.3 (1.3–1.3)	1.3 (1.3–1.3)	0.68
Baseline CD4 counts, mean (SD), cells/mm^3^	605 (229)	606 (305)	0.97
Baseline HCV RNA before treatment, median (IQR) log_10_ IU/ml	6.5 (5.6–7.1)	6.8 (6.3–7.1)	0.04
*Available results on the behavioural risk factors*			
“Slamming,” *n* (%)	19/111 (17.1)	3/27 (11.1)	0.57
Condomless receptive anal sex, *n* (%)	68/111 (61.3)	11/27 (40.7)	0.05
Condomless insertive anal sex, *n* (%)	59/111 (53.2)	14/27 (51.9)	0.90
“Rimming,” *n* (%)	39/111 (35.1)	11/27 (40.7)	0.59
Sharing dildos, *n* (%)	14/111 (12.6)	4/27 (14.8)	0.75
Having HIV-positive partners, *n* (%)	73/111 (65.8)	16/27 (59.3)	0.87

Notes: Recent-infection group comprised those who had seroconversion of anti-HCV antibodies after 2017 when DAAs were reimbursed by the National Health Insurance in Taiwan; or those who had new episodes of HCV viremia after 2017. Those who had HCV viremia before 2017 and those who had indeterminate chronicity of HCV viremia were classified as the remote-infection group. One hundred eleven patients (62.0%) in the recent-infection group and 27 (25.7%) in the remote-infection group participated in questionnaire interviews to inquire into the behavioural risk factors for acquiring HCV.

Abbreviations: HCV, hepatitis C; HBsAg, hepatitis B surface antigen; IQR, interquartile range; SD, standard deviation.

### Factors associated with HCV reinfection

Of all 34 cases of HCV reinfection, 32 (94.1%) occurred in MSM and 2 (5.9%) in injecting drug users (IDUs); and the incidence rates were 7.0 and 2.4 per 100 PYFU for MSM and IDUs, respectively, with an incidence rate ratio of 2.91 (95% CI, 0.7–25.1, *p *= 0.056).

To further understand the clinical and behavioural factors associated with HCV reinfection in the recent-infection group, we established two multivariate logistic models. The first model included all PLWH and assessed the impact of demographic and clinical factors, while the second focused on the behavioural factors and included only those who responded to the behavioural questionnaire interviews (Table 2). In the first model, the only demographic and clinical factors associated with HCV reinfection were recent syphilis (adjusted odds ratio [aOR], 2.73; 95% CI, 1.26–5.91) and age (aOR per 1-year increase, 0.95; 95% CI, 0.90–0.99).

**Table 2.** Multivariate analysis of factors associated with hepatitis C reinfections.

Risk factors	Univariate analysis, OR (95% CI)	Multivariate analysis, adjusted OR (95% CI)
*Model 1: demographic and clinical characteristics (n = 284)*		
Age, per 1-year increase	0.94 (0.90–0.99)	**0.95** (0.90–0.99)*
Injecting drug users	0.53 (0.12–2.35)	
Having received multiple (≥2) HCV treatments before this enrolment	0.29 (0.04–2.24)	
Recent syphilis within 3 months	3.09 (1.45–6.55)	**2.73** (1.26–5.91)*
Plasma HIV RNA at enrolment, per 1-log_10_ increase	0.22 (0.02–2.71)	0.16 (0.01–2.31)
CD4 cell count at enrolment, per 1-cell increase	1.00 (0.999–1.002)	
HBsAg positivity	0.69 (0.15–3.10)	
*Model 2: clinical characteristics and behavioural risk factors (n = 138)*		
Age, per 1-year increase	0.94 (0.90–0.99)	0.93 (0.86–1.02)
Recent syphilis within 3 months	3.09 (1.45–6.55)	2.74 (0.86–8.74)
Plasma HIV RNA at enrolment, per 1-log_10_ increase	0.22 (0.02–2.71)	
“Slamming”	1.15 (0.30–4.39)	
Condomless receptive anal sex	6.68 (1.46–30.5)	**14.5** (2.37–88.8)*
Condomless insertive anal sex	1.32 (0.47–3.68)	0.37 (0.10–1.42)
“Rimming”	2.20 (0.79–6.11)	**3.87** (1.14–13.1)*
Sharing dildos	0.38 (0.05–3.07)	0.17 (0.02–1.62)
Having HIV-positive partners,	0.91 (0.31–2.62)	

Abbreviations: OR, odd ratio; 95% CI, 95% confidence interval; HBsAg, hepatitis B surface antigen.

*Statistically significant in multivariate models.

In the second model, only 138 PLWH who participated in the behavioural questionnaire interviews (111 [62.0%] in the recent-infection group and 27 [25.7%] in the remote-infection group) were included. In the final model, only condomless receptive anal sex (aOR, 14.5; 95% CI, 2.37–88.8) and rimming (aOR, 3.87; 95% CI, 1.14–13.1) were linked to HCV reinfections among PLWH who had been successfully treated with DAAs.

### Evolution of HCV genotypes and phylogenetic analysis in HCV reinfections

**Figure 3.**
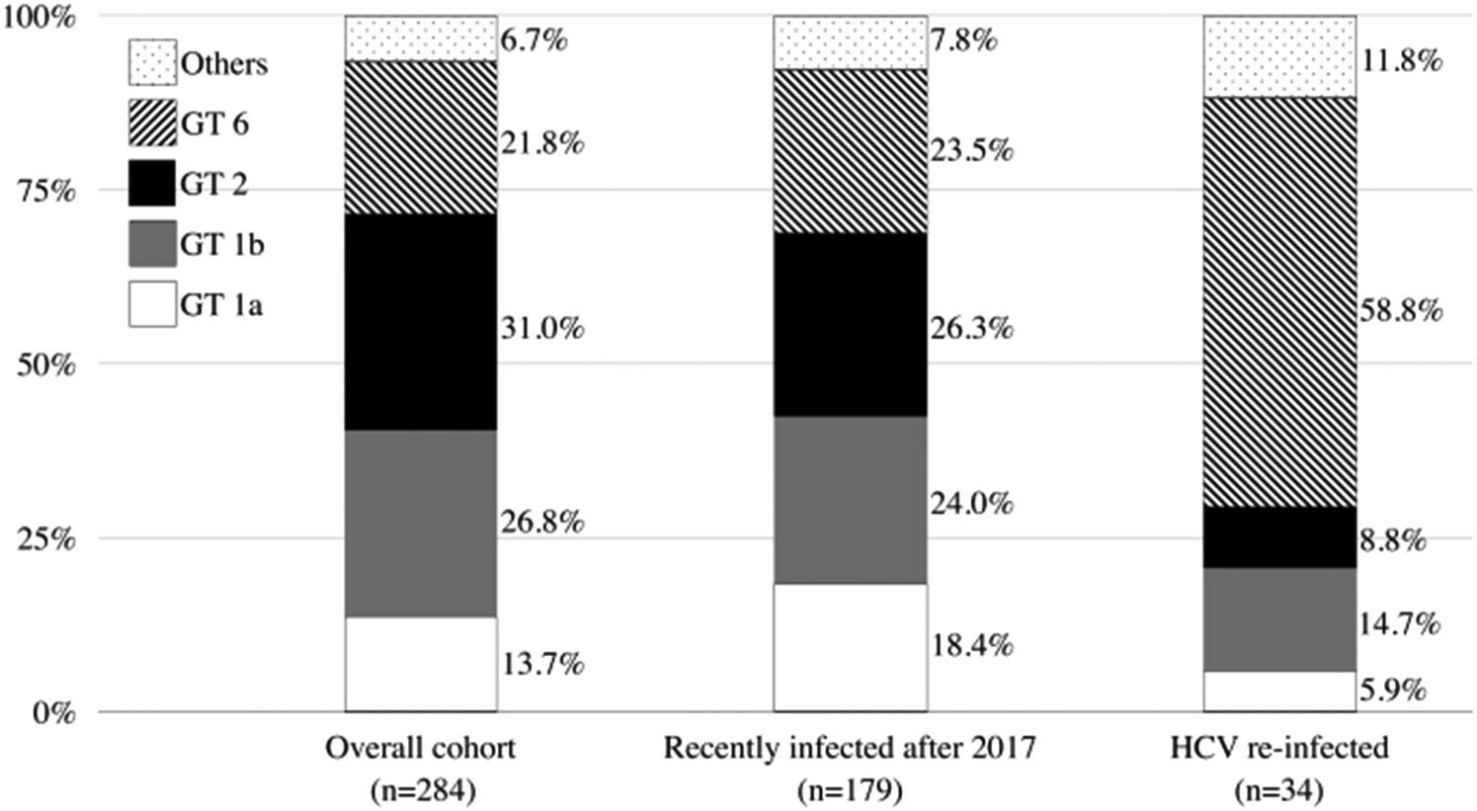
Evolution of HCV genotype distribution of the cohort. Abbreviations: GT, genotypes; HCV, hepatitis C virus.

**Figure 4.**
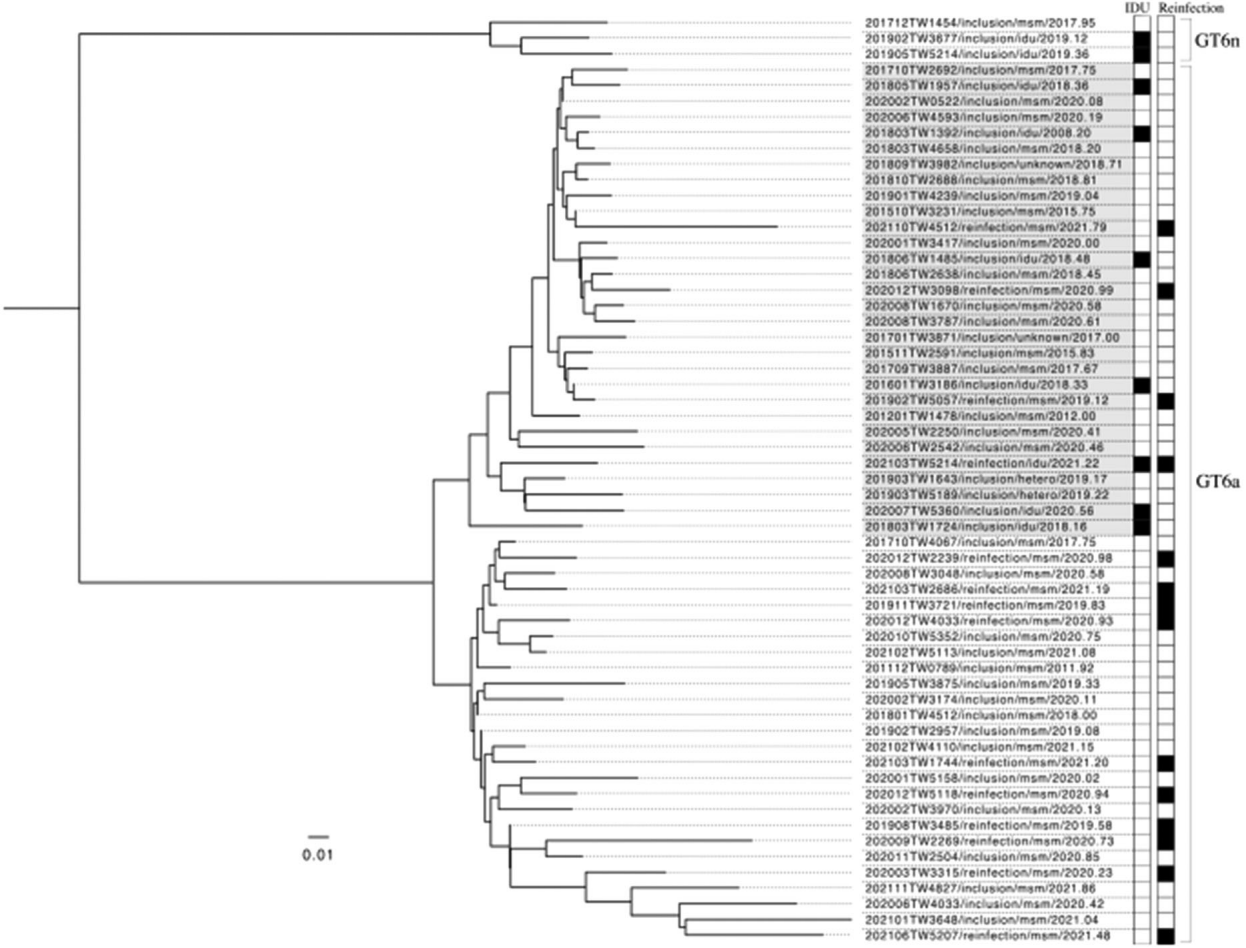
Phylogenetic analysis using *NS5B* gene sequences from 58 available HCV genotype 6 infections (44 samples collected from people living with HIV at the inclusion of this study and 14 from those with reinfection episodes) in our cohort. The results showed two major clades of HCV genotype 6a and a small number of genotype 6n infections in our cohort. HCV from injecting drug users (IDUs) and people living with HIV who had reinfections (reinfections) are marked black in the corresponding columns.

The distributions of HCV genotypes of the previous episodes of HCV viremia and recurrent episodes during follow-up are depicted in Figure 3. On inclusion into this study, the most commonly found HCV genotypes included genotypes 2 (31.0%), 1b (26.8%), 6 (21.8%), and 1a (13.7%). This distribution was similar between recent- and remote-infection groups. However, when HCV reinfections occurred, the proportion of genotype 6 significantly increased to 58.8% (20/34; *p *< 0.001), with the majority occurring in MSM (19 of 20 [95.0%] of genotype 6 reinfections). Furthermore, we successfully sequenced the HCV *NS5B* gene from 58 samples of HCV genotype 6 (44 from samples collected on study inclusion and 14 on reinfection episodes). We then performed a phylogenetic analysis with these available HCV sequences, and the results revealed two major clades of genotype 6a and a small number of genotype 6n infections. Interestingly most reinfections with genotype 6a belonged to the clade that was unrelated to those sequences of HCV genotype 6 identified from IDUs (Figure 4).

## Discussion

In this study, the overall rate of HCV reinfection was 5.8 per 100 PYFU (95% CI, 4.1–8.1) in the era of improved access to DAAs between 2018 and 2021, which was lower than that (8.2 per 100 PYFU; 95% CI, 5.2–13.1) observed in our previous study between 2011 and 2018, in which PLWH in interferon/ribavirin and early DAA eras were included [19]. However, we also demonstrated a significantly higher incidence rate of HCV reinfection (9.8 per 100 PYFU; 95% CI, 7.0–13.8) among PLWH who acquired HCV after 2017 (the recent-infection group) than that among those in the remote-infection group.

The difference in the incidence rates of HCV reinfection between these two groups should be interpreted with caution, as the baseline characteristics were not balanced. In our study, PLWH in the recent-infection group were significantly younger and more likely to be MSM and had a higher proportion of recent syphilis before the enrolment. It is expected that our recent- and remote-infection groups comprised different key populations of Taiwanese PLWH. Those in the recent-infection group were young MSM who were more likely to engage in risky sexual behaviours and thus were at higher risks of sexually transmitted infections (STIs), including HCV infection. The differences in baseline characteristics probably contributed to the significantly higher rate of HCV reinfection in the recent-infection group. The hypothesis is supported by the fact that, in multivariate analysis, HCV reinfection was linked to recent syphilis (aOR, 2.73; 95% CI, 1.26–5.91) and younger age (aOR per 1-year increase, 0.95; 95% CI, 0.90–0.99), but not injecting drug use (Table 2). Furthermore, HCV reinfection in our study was also associated with high-risk sexual practices such as condomless receptive anal intercourse (aOR, 14.5; 95% CI, 2.37–88.8) and rimming (aOR, 3.87; 95% CI, 1.14–13.1). Past studies have already demonstrated detectable HCV RNA in the semen and rectal fluid of HCV viremic patients [23,24], and mucosal breakage from concomitant STIs such as syphilis might facilitate the transmission of HCV.

The differences between PLWH in the recent-infection group and those in the remote-infection group imply how the epidemiology of HCV has evolved among Taiwanese PLWH. Traditionally, IDUs had been considered he highest risk of acquiring HCV among Taiwanese PLWH, especially from sharing syringes, needles or diluents [25,26]. However, HIV transmission among Taiwanese IDUs has drastically decreased with the sustained implementation of a harm reduction programme since 2005. In 2005, when the outbreak of HIV infection among IDUs reached its peak, 45.1% (1543/3422) of newly-diagnosed HIV cases reported to Taiwan Centers for Disease Control occurred among IDUs, which has significantly declined to 1.6% (17/1247) in 2021 [27]. Therefore, while PLWH in the recent-infection group, mostly high-risk MSM continued their risky sexual behaviours and were at higher risk for HCV reinfections, the risk for HCV reinfections after DAA treatment among IDUs who were remotely infected with HCV and engaged in HIV and HCV care had significantly reduced.

Our study is the first to observe the expansion of HCV genotype 6 among PLWH who were MSM and had HCV reinfections. In previous studies, HCV genotype 6 infections mainly occurred in IDUs in Taiwan and several Asian-Pacific countries [28–30], but our new findings suggested HCV genotype 6 might have circulated among MSM with high-risk sexual practices. In Taiwan, studies before 2005 universally demonstrated a very low prevalence of HCV genotype 6 in the general population [31,32]. After the outbreak of HIV and HCV among IDUs in 2005, studies have shown that the seroprevalence of HCV genotype 6 had increased to 5%–10% in the general population [33,34]. In terms of PLWH, the prevalence of HCV genotype 6 was around 20%–30%, which also was likely associated with IDUs who had HIV coinfection [35–37]. However, results from our phylogenetic analysis revealed that most reinfections with genotype 6a were linked to the clade that was unrelated to those HCV *NS5B* sequences from IDUs (Figure 4). With our observation, we are concerned that the key demographics of HCV genotype 6 infection might have shifted from IDUs to MSM in Taiwan. The exact routes by how genotype 6 had spread from Taiwanese IDUs to the MSM population remain speculative. Possibly, there were individuals who shared these two risks (condomless anal intercourse and injection drug use) for HIV and HCV transmission and served as the bridge of transmitting genotype 6 from one population to another. Recently, the increase of “slamming” and “chemsex” among the MSM community in Taiwan have raised significant concerns. In our behavioural questionnaire interviews, around 20% of the participants reported experiences of “slamming” during parties or dates. Limited by our small case number, this speculation warrants confirmation by more clinical and epidemiologic studies, in Taiwan and other Asian-Pacific countries, as this region is frequently considered the epidemic centre of HCV genotype 6 worldwide.

Our study had several limitations, and our findings should be interpreted with necessary caution. Firstly, our study focused on HIV-positive MSM who had been receiving HIV care in our hospital, and the results could only be better explained under the context of HIV/HCV epidemiology in Taiwan. The difference in the incidences of HCV reinfection observed between the recent- and remote-infection groups might not be applicable in other populations or countries. Therefore, our findings may not be generalizable to key people in other geographic regions. Our findings highlighted the importance of epidemiological studies at the local level, which might improve our understanding of HCV epidemiology and inform the policies to achieve HCV elimination. Secondly, the proportion of participation in the pooled-plasma HCV RNA testing study and behavioural questionnaires was not balanced between the two groups. In the recent-infection group, 62.0% of included PLWH participated in the pooled-plasma HCV RNA testing study and responded to the behavioural questionnaires, as compared to only 25.7% in the remote infection group. Therefore, the interpretation of risk factors in the remote-infection group should be cautious as they were under-represented. Moreover, as the participants in the pooled-plasma HCV RNA testing study tested for HCV viremia regularly every 3 months, the incidence rate in the remote-infection group might be underestimated due to the lower testing frequency. Furthermore, since using illicit drugs is a criminal offense in Taiwan, IDUs who had been remotely infected with HCV and were able to attend our clinics regularly might be at an even lower risk of repeating their risk behaviours. This selection bias might also lead to underestimating the reinfection rate among IDUs in our study.

Our study found a high rate of HCV reinfection among PLWH who acquired HCV after 2017, compared with those whose HCV infection occurred before 2017. Instead of injecting drug use, these episodes of HCV reinfections were associated with high-risk sexual behaviours among MSM. The predominance of HCV genotype 6 reinfections suggested on-going clustered HCV infections among at-risk PLWH.
